# Development of *In Vitro* Methodologies to Investigate Binding by Sodium Hyaluronate in Eye Drops to Corneal Surfaces

**DOI:** 10.2174/1874364101812010226

**Published:** 2018-07-31

**Authors:** Udo Bock, Von Deylen D, Jochner M, Doerr M, Stäbler C, Reichl S

**Affiliations:** 1 Bock Project Management, Tawern, Germany; 2 Technische Universität Carolo-Wilhelmina zu Braunschweig, Institut für Pharmazeutische Technologie, Braunschweig, Germany; 3 Bayer Vital GmbH, Scientific Affairs Consumer Health, Leverkusen, Germany

**Keywords:** Sodium Hyaluronate, Cornea, Binding, Residence Time, Eye drops, HCE-T

## Abstract

**Purpose:**

To develop *in vitro* methods to assess binding by sodium hyaluronate in eye drops to corneal surfaces.

**Methods:**

Two different, complementary corneal binding set-ups were developed. In a dynamic *in vitro* model, confluent corneal epithelial cells (HCE-T) were assembled in chamber slides and a declining channel. A static model was constructed with *ex vivo* porcine corneas clamped in Franz cells. To test the predictive capacity of models, four different eye drops containing sodium hyaluronate were spiked with tritium-labeled sodium hyaluronate to standardize quantification. In both settings, eye drops were applied for 5 min and physiological conditions were mimicked by flushing with artificial tear fluid. Spreading experiments on HCE-T next to synthetic membranes were used for further characterization.

**Results:**

Binding was more pronounced in dynamic HCE-T model. Three of the four eye drops demonstrated sigmoidal elution of sodium hyaluronate, suggesting pronounced binding. One solution eluted distinctly faster, likewise the buffer control. The static method produced a similar ranking but at lower levels. When eye drops in which phosphate buffer was replaced by citrate buffer (*i.e.*, to prevent calcification) were used, binding was not influenced. All eye drops spread immediately when placed on HCE-T and at the same order of magnitude on glass and polyethylene terephthalate surfaces.

**Conclusion:**

Dynamic and static models performed on different corneal sources were used to determine sodium hyaluronate binding kinetics in solutions under physiological conditions. These methodologies resulted in a ranking of the capacity of sodium hyaluronate to bind *in vitro* to corneal surfaces.

## 1 INTRODUCTION

Dry Eye Disease (DED) is a common condition around the world that affects up to one in every three people [1]. Although DED can occur in people of any age, the risk of developing DED increases with age, and it is more common in women than in men [2]. Symptoms include ocular irritation, pain and transient visual impairment, mostly due to tear film instability or a change in its composition that leads to a disruption in the tear film [3]. Usually, both eyes are affected. The symptomatic therapy of choice is supplementation with artificial tears to lubricate the eye, replace missing tear fluid and normalize tear film osmolarity [4]. The naturally occurring endogenous factor hyaluronate induces long-lasting, intensive ocular lubrication [5], and this explains why several artificial tear solutions contain sodium hyaluronate (Na-HA) as a moisturizing and lubricating substance. However, commercial eye drops contain different amounts of hyaluronate and different types of buffers (*i.e.*, phosphate or citrate buffer) and differ in their rheology and additives. Additionally, most molecular weights of the hyaluronates that are incorporated into eye drops vary or are unknown.

The purpose of these *in vitro* studies was to develop methodologies that can be used to assess the magnitude and binding kinetics of Na-HA in eye drops used on corneal surfaces and to compare adhesion among the respective eye drops in two different settings. Representative commercial eye drops with different compositions were selected and design-supplemented by buffer control. All of the selected eye drops contained Na-HA but in different amounts. The amounts ranged from 0.10% Na-HA in HYLO-COMOD^®^ and Hyaluron-ratiopharm^®^ eye drops to 0.15% in Bepanthen^®^ eye drops and BLUpan^®^ medical UD. To standardize and allocate *in vitro* binding by sodium hyaluronate, the methods were designed to take into account elaborated corneal surfaces, including monolayers of cultured Human Corneal Epithelial Cells (HCE-T) and *ex vivo* porcine corneas. Both *in vitro* corneal settings have been characterized in detail regarding their corneal permeation [6, 7] and metabolism [8], and these results have been correlated with those observed in the human cornea [9]. Lacrimation is a physiological condition that is included in both, the static Franz diffusion cell (porcine cornea) model and the declining channel set-up (HCE-T) model. In both, flushing with artificial tear fluid was used.

## 2 MATERIALS AND METHODOLOGY

### 2.1 Materials

#### 2.1.1 Eye Drops

The following four different eye drops that contain Na-HA and are available on the German market were selected: HYLO-COMOD^®^ (Ursapharm Arzneimittel GmbH, Saarbrücken, Germany) [10], Hyaluron-ratiopharm^®^ eye drops (Ratiopharm GmbH, Ulm, Germany) [11], Bepanthen^®^ eye drops (Bayer Vital GmbH, Leverkusen, Germany) [12], and BLUpan^®^ medical UD (Pharma Stulln GmbH, Stulln, Germany) [13]. Phosphate-buffered saline (PBS) was used as the control. HYLO-COMOD^®^ contains 1 mg/mL sodium hyaluronate, citrate buffer, sorbitol and water. Hyaluron-ratiopharm^®^ eye drops contain 1 mg/mL sodium hyaluronate, sodium chloride, sodium hydrogen phosphate, chloride (potassium, calcium, and magnesium), sodium hydrogen carbonate, and water. Bepanthen^®^ eye drops contain 0.15% sodium hyaluronate, 2% panthenol, sodium chloride, sodium monohydrogen phosphate, sodium dihydrogen phosphate, and water. BLUpan^®^ medical UD contains 0.15% sodium hyaluronate, 2% panthenol, sodium chloride, sodium citrate, citric acid, and water.

#### 2.1.2 3H-Sodium Hyaluronate (3H-Na-HA)

Hyaluronic acid [3H(G)] sodium salt (MW 300.000 Da) with a specific activity (assay January 2016) of 260 mCi/g in sterile water was obtained from American Radiolabeled Chemicals Inc., USA. Material was stored at recommended temperature of 0-5°C and used within 6 months. Each spiking was performed immediately before start of the experiments.

#### 2.1.3 Artificial Tear Fluid

The tear fluid used in this study was composed of 0.68% sodium chloride, 0.05% potassium chloride, 0.014% sodium dihydrogen phosphate monohydrate, 0.21% sodium bicarbonate, 0.36% HEPES, 0.11% D-Glucose monohydrate, 0.02% magnesium sulfate heptahydrate, 0.01% calcium chloride dihydrate and lysozyme extracted from human milk powder (1.2 g/L). All substances (except lysozyme) were dissolved in double-distilled water. After the solution was equilibrated to room temperature, lysozyme was added, and the pH was adjusted to 7.4 with NaOH or HCl. The artificial tear fluid (ATF) was then sterile-filtered and stored at -20°C until used.

#### 2.1.4 Porcine Cornea in Franz Cells (FC)

An assessment of *in vitro* adhesion was performed using an *ex vivo* model of porcine corneas and FC. Porcine eyes were obtained from a local slaughterhouse and stored in sterile PBS until used. The corneas were excised and immediately mounted in FC (incubation area per cornea: approx. 0.64 cm^2^).

#### 2.1.5 HCE-T Cell Monolayer on Chamber Slides

Cell monolayers composed of human corneal epithelial cells were used. SV40-transfected Human Corneal Epithelial Cells (HCE-T cells) [14] were cultivated to form monolayers according to the methods described in earlier studies [15, 16]. *In vitro* adhesion studies were performed with HCE-T monolayers on Chamber Slides (CS). The dimensions were 50 mm long and 18 mm wide (including a 9 cm^2^ growth area for the monolayer). The following were the total dimensions of the CS: 76 mm x 24 mm (Sarstedt AG & Co., Germany). For details, see Fig. (**1**).

### 2.2 Methodology

#### 2.2.1 Incorporation of 3H-Na-HA into Eye Drops and PBS

As no information is currently available regarding the molecular weights of the Na-HA in each of the different products used in this study, tritium-labeled Na-HA (3H-Na-HA) was incorporated into the eye drops to facilitate a direct comparison of the effects of the formulations on adhesion. Moreover, an assessment of 3H labeling allowed us to perform an analytical quantification of *in vitro* adhesion *via *scintillation counting. All products were used unaltered, and all flasks were opened immediately before they were spiked with 3H-Na-HA for *in vitro* tests. The final levels of radioactivity observed in the FC experiments were 0.5 µCi/mL and 1.0 µCi/mL performed in the declining Channel (dC) set-up.

#### 2.2.2 Adhesion on *Ex Vivo* Porcine Cornea in FC

Immediately after the corneas were clamped into FC, the surfaces were incubated with 3H-Na-HA eye drops or PBS (a 400 µL volume was spread equally over the whole surface), and the corneas were incubated for 5 min while gently rotating at 100 rpm. A stepwise dilution with ATF was then performed for 10 min as follows. Every minute, 200 μl of a donor solution was replaced with an equal amount of ATF. The amount of 3H-Na-HA in the donor solution was determined both before and after incubation in addition to after dilution. The amount of 3H-Na-HA on the corneal surfaces was determined after the experiments ended. All studies were conducted in triplicate (*n* = 3).

#### 2.2.3 Adhesion to *in Vitro* HCE-T Monolayers in the Declining Channel Model

**Fig. (1):**
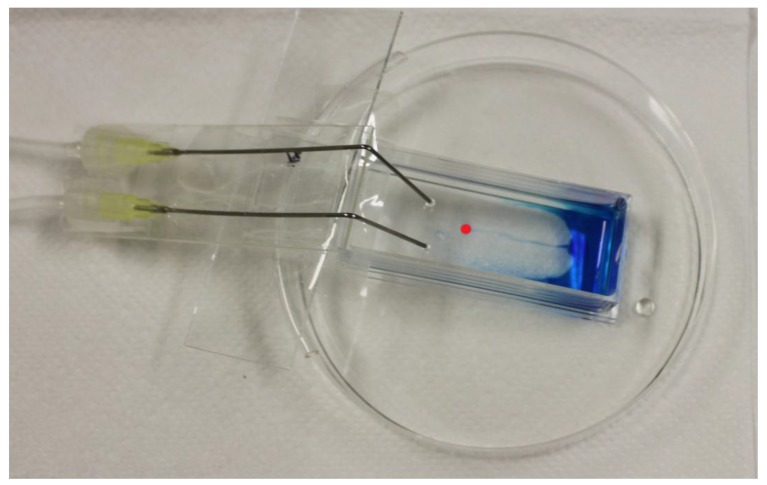
Method development with dye methylene blue on HCE-T monolayers grown on CS. Dye (0.1%) was used as a surrogate to determine experimental parameters on angle, number of needles, flow rates and elution zones on CS. Final parameters fixed on *in vitro* adhesion testing: declining channel at 11° and flow rate of ATF at 18 µL/min. A 35 µL sample of dye (equal later to one drop of ED) was placed between two needles, indicated herein by red dot. The picture was taken at approx. 1 min, demonstrating optimized elution zones. Within the total period of 5 min, most of the dye was eluted at the 1 min time point, confirming negligible binding.

In the dynamic study design, we used the dC model and *in vitro* HCE-T monolayers grown on CS. The dC concept was first introduced for mucosal tissues [17] and was later developed to evaluate mucoadhesion on corneas [18, 19]. The elution of ATF was performed using a KDS270 Continuous Cycling Syringe Pump (KD Scientific, Holliston, Massachusetts, USA). The final flow rate was set to 18 µL/min, in accordance with the *in vivo* human tear fluid turnout rate [20, 21]. In preliminary experiments performed to evaluate binding by 3H-Na-HA in ED, methylene blue (0.1%) was used as a surrogate to determine the experimental parameters, including the angle, number of needles, flow rates and elution zones (Fig. **1**). This blue dye allowed the direct visualization of flow characteristics. The flow rate was initially started at 0.6 to 0.8 mL/min and was decreased to a 33- to 44-fold lower rate of 18 µL/min. Finally, it was necessary to use two needles to avoid the formation of “undiluted” zones at the edges of the CS. CS with HCE-T monolayers were placed on dC at an angle of approximately 11°. The angle was determined based on the height of a petri dish (15 mm) and the total length of the chamber slide (76 mm). A defined volume of 35 µL of 3H-Na-HA-spiked eye drops or PBS was applied to the upper end of the CS (between the two needles) containing HCE-T monolayers. The 35 µL volume (corresponding to approximately one ED drop) was applied while the CS was already at 11° and 250 µL buffer was applied at the bottom. In accordance with the study design used in the FC/*ex vivo* porcine cornea experiments, the duration of exposure was fixed at 5 min. All studies were conducted six times (*n* = 6).

#### 2.2.4 Spreading

The process of spreading the cells on the corneal surfaces prior to the experiments was uncoupled from the static and dynamic corneal evaluations and investigated by measuring the contact angles of the different surfaces. The dynamic contact angles of the eye drops were measured on membranes using a G10 contact angle analyzer (Krüss GmbH, Hamburg, Germany) equipped with an axisymmetric drop shape analysis profile. The surfaces exhibited varying physical and biological properties. Glass (hydrophilic) and polyethylene terephthalate (PET, hydrophobic) had solely physical characteristics. Cell monolayers of HCE-T cells were grown on chamber slides and used to evaluate biopharmaceutical relevance. The eye drops were equilibrated to room temperature at least 24 h before these experiments were performed. During measurement, whole-drop geometry was recorded and mathematically fitted. Fitting of the contact angles was determined for solid-to-liquid boundary surfaces. All eye drops used in the determinations were used unaltered and were immediately opened before *in vitro* tests were performed (each experiment was performed six times).

#### 2.2.5 Scintillation Counting

*In vitro* samples (200 µL FC or 100 μl dC) were transferred into 2000 µL scintillation cocktails and measured on an LS-6500 Liquid Scintillation Counter (Beckman Coulter, Brea, California, USA).

#### 2.2.6 Statistical Analysis

The data are shown as the mean ± Standard Deviation (SD). For each experimental setting, k*(*k*-1)/2 pairwise comparisons were performed in which *k* represented the number of the solution being investigated. The results were tested using Dunn’s test. All *p-*values were adjusted using the Bonferroni correction. Only corrected *p* values less than 0.05 were considered statistically significant. Computations were carried out using R and the PMCMR package.

## 3 RESULTS

### 3.1 Binding to Porcine Cornea *Ex Vivo*

**Fig. (2):**
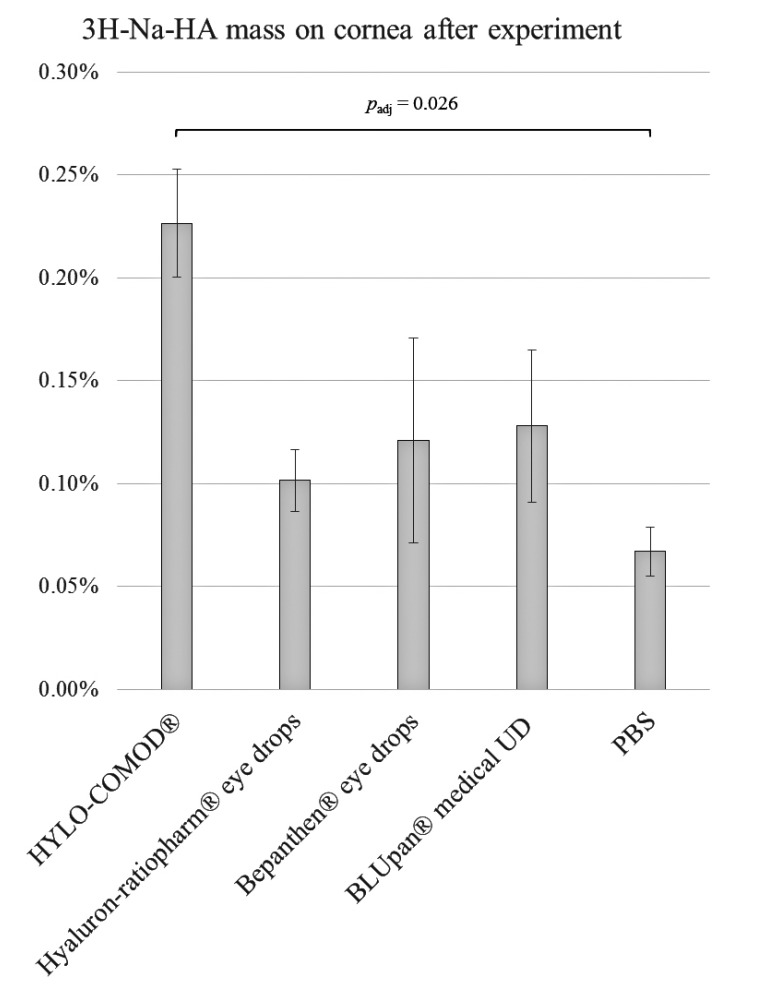
Comparison of binding of 3H-Na-HA within the *in vitro* assay porcine cornea and FC. Percentages are calculated on initial activity of 3H-Na-HA at start. Data are mean values [%] ± SD [%] from experiments performed in triplicate. Significant different pairs (*p*_adj_ < 0.05) are indicated with *p*_adj_ value.

In the FC setting, only a small amount of 3H-Na-HA (close to the quantification limit) adhered to the *ex vivo* porcine corneas (Fig. **2**). With regard to the amount of 3H-Na-HA that was applied at the start of the experiment, all of the tested eye drop formulations had comparable retention rates, as follows: 0.23% ± 0.03% for HYLO-COMOD^®^, 0.10% ± 0.02% for Hyaluron-ratiopharm^®^ eye drops, 0.12% ± 0.05% for Bepanthen^®^ eye drops, and 0.13% ± 0.04% for BLUpan^®^ medical UD. In comparison, a considerably lower amount of 3H-Na-HA was obtained from the PBS-treated corneas (0.07% ± 0.01%). This amount was close to the quantification limit of the scintillation method. The total mass balance after the experiment was in the range of 81-102%, indicating a complete recovery. Although the data plots appeared to indicate that there were differences between the solutions, only HYLO-COMOD^®^ was found to be significantly different from PBS (*p*_adj_ ≈ 0.026).

### 3.2 Binding on HCE-T Monolayer *In Vitro*

**Fig. (3):**
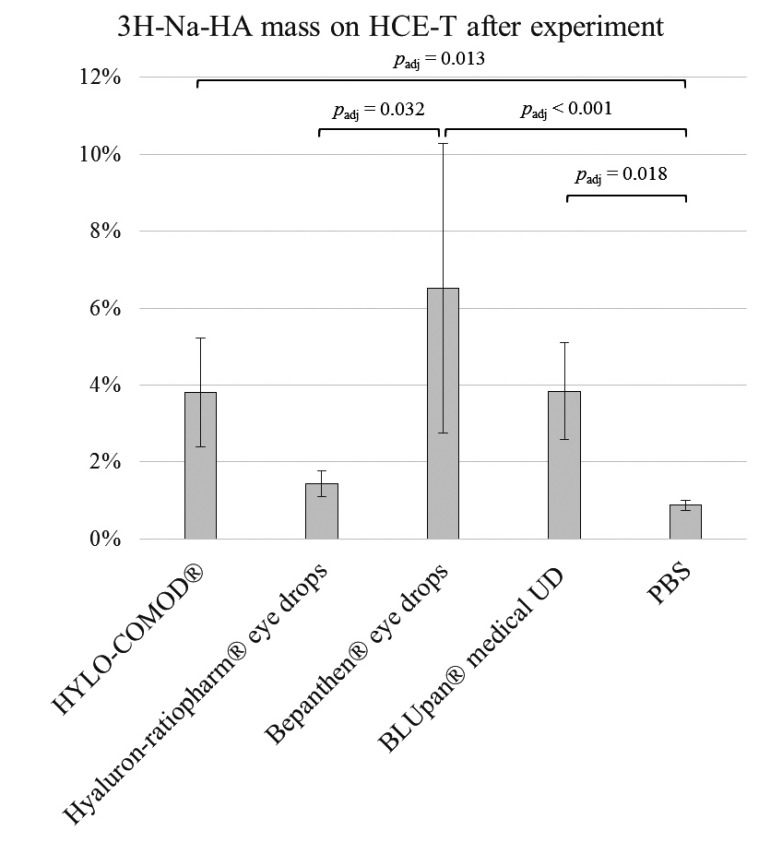
Comparison of binding of 3H-Na-HA within the *in vitro* assay HCE-T and dC. Percentages are calculated on initial activity of 3H-Na-HA at start. Data are mean values [%] ± SD [%] from experiments performed in in six-fold. Significant different pairs (*p*_adj_ < 0.05) are indicated with *p*_adj_ value.

Consistent with the FC and porcine cornea experiments, the results of the declining channel set-up of HCE-T monolayers grown on CS showed that the retention rates were 3.8% ± 1.4% for HYLO-COMOD^®^, 1.4% ± 0.3% for Hyaluron-ratiopharm^®^ eye drops, 6.5% ± 3.8% for Bepanthen^®^ eye drops and 3.8% ± 1.3% for BLUpan^®^ medical UD. This order was similar to that observed for 3H-Na-HA binding by the respective eye drops. As in the FC setting, the amount of 3H-Na-HA that was obtained from PBS (0.9% ± 0.1%) was considerably lower (Fig. **3**). The total mass balance after the experiment was in the range of 87-110%, indicating a complete recovery. A statistical comparison of the Bepanthen^®^ eye drops, the BLUpan^®^ medical UD (*p*_adj_ = 1) and HYLO-COMOD^®^ (*p*_adj_ = 1) showed that there were no differences among these treatments. However, there was a significant difference between the Hyaluron-ratiopharm^®^ eye drops (*p*_adj_ ≈ 0.032) and the PBS control (*p*_adj_ < 0.001).

**Fig. (4):**
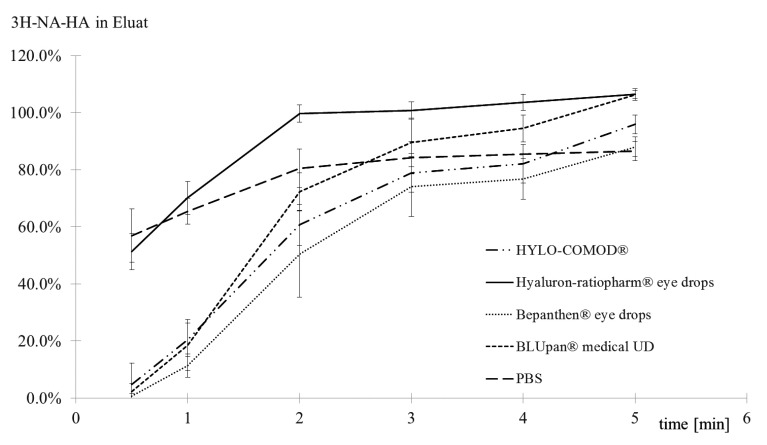
Kinetics of elution of 3H-Na-HA in % of dose applied within the *in vitro* assay dC and HCE-T. Percentages are calculated on initial activity of 3H-Na-HA at start. Data are mean values [%] ± SD [%], *n* = 6.

An analysis of the elution kinetics (Fig. **4**) of 3H-Na-HA revealed that there were differences between the tested products. While HYLO-COMOD^®^, Bepanthen^®^ eye drops and BLUpan^®^ medical UD exhibited a sigmoidal increase in 3H-Na-HA over time, Hyaluron-ratiopharm^®^ eye drops eluted distinctly faster in a pattern similar to that of PBS.

With regard to the difference in buffer between Bepanthen^®^ eye drops (phosphate buffer) and BLUpan^®^ medical UD (citrate buffer), no significant difference was confirmed (*i.e.*, the exchange did not impact the binding of 3H-Na-HA).

#### 3.3 Spreading

**Fig. (5):**
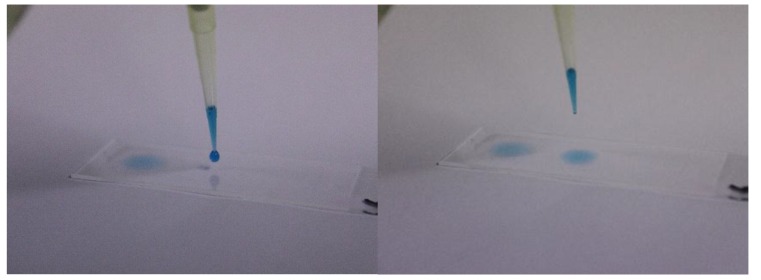
Representative picture of spotting a product stained with dye methylene blue (0.1%) on a HCT-monolayer. Left picture: blue spot: first drop applied and second drop immediately before application. Right picture: the second drop spread immediately over the HCE-T monolayer surface.

The contact angles (Table **1**) measured on hydrophobic PET surfaces were pronounced, whereas the angles on HCE-T monolayers were negligible and allowed complete spreading. Spreading on glass surface was in-between HCE-T and PET. For all products, the mean contact angles on glass surfaces ranged from 42.9° to 48.9°, while those on PET surfaces ranged from 105.7° to 112.1°. In each subgroup of glass and PET surfaces, the contact angles were within the same order of magnitude. A statistical analysis of Bepanthen^®^ eye drops *versus* BLUpan^®^ medical UD showed that there was no difference in the contact angles they produced on glass (*p*_adj_ = 1) or PET surfaces (*p*_adj_ = 1), indicating that the switch from phosphate buffer in Bepanthen^®^ eye drops to citrate buffer in BLUpan^®^ medical UD had no impact on spreading. While no statistically significant differences were found for glass, spreading on PET was significantly different between the Hyaluron-ratiopharm^®^ eye drops and the Bepanthen^®^ eye drops (*p*_adj_ ≈ 0.002) and BLUpan^®^ medical UD (*p*_adj_ ≈ 0.017). On the biological surface (*i.e.*, the HCE-T monolayer), all of the eye drops spread over the surface immediately after the drop hit the surface (Fig. **5**). Adjusted p-values for all pairwise comparisons between contact angles for glass and PET surface were summarized (Table **2**).

## 4 DISCUSSION

When developing eye drop fluids to adequately bind to the cornea, the physiological conditions of the corneal surface and its different layers must be taken into account. For example, researchers must consider the pronounced structure of the tear film that covers the ocular surface. Insufficient tear film production or a change in its composition can disrupt the tear film. As a direct consequence of such a disruption, oxygen and nutrients are withdrawn from the cells of the ocular surface, leading to potential cellular damage that can, in turn, cause the characteristic symptoms of DED.

A comprehensive three-part classification framework of DED summarized [22, 23] the etiopathogenic and multiple causes of dry eye, the mechanistic pathway of disease (*i.e.* Sjogren and Non-Sogren syndrome dry eye, evaporative dry eye, tear hyperosmolarity and tear film instability) and severity gradings. Tear Film and Ocular Surface Society (TFOS) updated an evidence-based definition and a contemporary classification system for DED [24], which consider the multifactorial nature of disease. A central pathophysiological aspect is attributed to the loss of homeostasis of the tear film. State of the art diagnose and monitoring of DED, appropriate order and techniques in clinical settings and subclassification were subject of TFOS diagnostic methodology report [25]. Pathophysiology of DED [26, 27] describes dry eye as a chronic inflammatory disease, which is based on numerous extrinsic or intrinsic factors that promote unstable and hyperosmolar tear film. Changes in the composition in combination with systemic factors could start an inflammatory cycle, which is accompanied by ocular surface epithelial disease and neural stimulation. Activation of stress signaling pathways in the ocular surface epithelium and resident immune cells induce production of innate inflammatory mediators. Imbalance in the protective immunoregulatory and proinflammatory pathways of the ocular surface summarize dry eye as a mucosal autoimmune disease [28, 29]. An anatomy based extension of ocular surface and immunology view [30] included the lacrimal gland [31] and the lacrimal drainage system, which result in drainage of tears and ocular surface integrity. Overall, the immune system of the ocular surface forms an eye-associated lymphoid tissue and component of the mucosal immune system. As a consequence, ocular surface was considered as compartment of the common immune system [32].

The comprehension of normal tear film [33] and importance in DED resulted in numerous approaches to assess ocular diseases *via *proteomics and metabolomics of tear film [34, 35], biomarkers [36-38] or selected metalloproteinases [39]. Changes of tear film in DED and association to ocular surface [40] accentuated knowledge of the different layers of the tear film including constituent parts. An aqueous tear film covers human cornea and is typically divided into three layers, which could be easily distinguished as mucin layer (2.5 to 5 μm thickness) with contact to corneal epithelium, an aqueous layer (approx. 4-μm) and a relatively thin lipid layer (0.015 to 0.160 μm) dividing it from the external environment [41]. Three key processes of tear flow, evaporation and blinking influence the non-static and inhomogeneous film. Dynamics of tear flow and evaporation could assemble a stationary process, whereas blinking is a non-regular disturbance of the tear film. Duration of spontaneous eyelid down- and up-movement is approximately 100 ms to 250 ms.

The mucin layer (first layer) is directly located at the corneal surface and anchored to the epithelium. Epithelial cells produce the main component of sugar-rich glycosylated proteins and form a gel-like structure. This easily wettable surface layer assist in water re-spreading after blinks [41]. Different mucins are distributed on the ocular surface [42]. Part of the mucins are secreted by goblet cells and soluble in the tear fluid, a further part of membrane-associated classes form a dense barrier in the glycocalyx at the epithelial tear film interface [43]. The second aqueous layer contains next to aqueous phase numerous water soluble and insoluble components (electrolytes, soluble mucins, proteins, peptides and small molecule metabolites). Proteomics of tear film assigned most abundant proteins lipocalin (approx. 2 mg/mL) and lysozyme (approx. 2.5 mg/L). Amphiphilic and surface active lipocalin [44] is supposed to assist in tear film spreading, lysozyme [45] is justified by its high antimicrobial activity next to further antimicrobial compounds in tears [46]. The aqueous layer offers lubrication during blinks and eye movements, prevention of eye surface dehydration, protection against pathogens and small particles from air, and nutrition of corneal cells. Lacrimal glands continuously secret at a flow rate of approx. 1.2 μL/minute the fluid across the eye surface and removal *via *nasolacrimal duct. The outermost third part of the tear film is a thin layer of lipids. This lipid layer reduces the surface tension of film and re-spreading after blinking. Lipid movement and film reconstruction is relatively fast and driven *via *a concentration gradient of polar lipids. Within tear film break-up, the film thickness will reduce until collapse. In humans, time is in-between seconds and up to one minute. In DED period might be further reduced to few seconds or the breakup can even occur instantaneously after a blink [41]. Meibomian gland dysfunction and change in Tear Film Lipid Layer (TFLL) composition is known as a leading cause of dry eye syndrome [47]. Accordingly, the composition, structure and function of TFLL were intensively investigated starting with mapping of lipidome [48, 49] and molecular organization [50].

To overcome an impaired fluid layer, Na-HA is ideally suited because it displays a long-lasting, intensive ocular lubricant [5]. Additionally, the chemical structure of hyaluronate allows it to retain water up to many times of its own weight. Importantly, hyaluronate adheres to the ocular surface, on which it forms a uniform and stable film that keeps the eyes lubricated. Today, a multitude of eye drops are available that contain Na-HA [51]. A recent summary of the rheological properties of these eye drops classified them into five different categories based on their increasing viscosity [52]. While HYLO-COMOD^®^ and Bepanthen^®^ eye drops were assigned to category three, Hyaluron-ratiopharm® and BLUpan® medical UD were unfortunately not included in this survey. These rheological groups were in agreement with the outcomes of the present *in vitro* corneal binding experiments.

A total of three major approaches have been implemented during the years that allow Corneal Residence Times (CRT) to be determined and visualized *in vitro* and *in vivo*. In many cases, the depth of application of Na-HA was the focus of these studies. Chronologically, the first investigations used gamma scintigraphy [53-57] with sodium pertechnetate Tc-99 m. In the majority of such studies, this technique was applied in healthy volunteers and/or sicca patients to explore the effects of varying concentrations of Na-HA [53-55]. Alternative polymers, such as HPMC or PVA, have also been used with Na-HA to enhance CRT [55-57]. In accordance with our *in vitro* findings, the CRT of Na-HA was rather short (approximately 3-10 min) but superior to that of the PBS control. Additionally, Na-HA CRT was not different between healthy and keratoconjunctiva sicca groups, and increasing Na-HA concentrations led to increased binding. Finally, the impact of 0.2% Na-HA on CRT was superior to that of 0.3% HPMC and 1.4% PVA [56]. A second test strategy for CRT involves analyzing tear fluid to explore the effects of active drugs [58-60], *i.e.*, *via *HPLC. Dependencies that affect CRT have mostly been studied in rabbits, in which different polymers, including Na-HA [58], nanoparticles [59] and mixtures of polymers containing Na-HA [60] have been used. Finally, combining fluorescent labeling of mucoadhesive polymers and confocal microscopy have enabled the sensitive measurement and visualization of CRT [61-65]. An improved microscopic *in vivo* method that has been used in rabbits and humans involves the use of fluorimetry measurements to determine the precorneal residence time of novel formulations containing non-penetrating FITC dextran (MW 70-73 kDa) [61]. This technique enabled the time to return to baseline to be recorded and AUC and T_50_ values to be determined. In another study, fluorescein sodium dissolved in 0.1% HA solution and 0.1% fluorescein-conjugated with hyaluronic acid (FHA) dissolved in saline were instilled into the eyes of healthy volunteers [62]. After 10 min, the turnover rates of the 0.1% FHA, 0.1% HA and saline solutions were 8.1%/min, 21.6%/ min, and 31.0%/min, respectively, demonstrating that HA has a prolonged retention time on the ocular surface. Fluorescence assays have applied doxorubicin due to its intrinsic fluorescence to deliver drugs, including liposomal formulations [63], mucoadhesive polymers [64] and active drugs, such as timolol [65], and these studies have achieved results consistent with those obtained using gamma scintigraphy and active drug determinations. Finally, the results of our *in vitro* binding experiments aimed at exploring Na-HA CRT are in line with and showed similar periods to FHA studies and showed that Na-HA was superior to the buffer controls.

The results of *in vitro* binding experiments confirmed the results of *in vivo* experiments, performed using currently available techniques, with regard to the grading and extent of Na-HA binding that was achieved. In general, the use of tritium-labeled Na-HA allowed us to combine the sensitivity of gamma scintigraphy and fluorescence assays with the direct detection of Na-HA. This was similarly true for the tear fluid assays performed without structural drug modifications. Hence, our simplified method produces results faster than later *in vivo* studies and allows the ability to establish predictiveness and selected physiological conditions and to make *in vivo* correlations. Additionally, this method can be quickly implemented. A retrospective inspection of the Na-HA CRT data illustrated further validation parameters relevant to CRT, such as different drug delivery strategies, and their effects on Na-HA. These include adhesive polymers, nanoscale formulations and dose-response curves for Na-HA. Moreover, our *in vitro* binding assays avoid the time spent during the early stages of development in addition to avoiding the cost-consuming animal and human trials. The clinical data on keratoconjunctiva sicca patients [54, 55] showed that the implementation of *in vitro* CRT binding in an *in vitro* disease model might be a future optimization step. Finally, the combination of permeability assays and CRT binding was introduced for use with HCE-T monolayers [66].

In our studies, regardless of whether a static or dynamic *in vitro* model was used, the binding behaviors of Na-HA in HYLO-COMOD^®^ and Bepanthen^®^ eye drops were comparable. Furthermore, their binding kinetics had similar profiles. We used *in vitro* models (static *versus* dynamic) independently to assess the adhesion rates and binding kinetics of Na-HA on the ocular surface under physiological conditions by controlling the amount of eye drop fluids that were applied or how much tear fluid volume exchange occurred. These analyses showed that similar results were obtained with regard to how the formulations were ranked in both *in vitro* settings. Binding effects were more pronounced in the HCE-T model. In both set-ups, Na-HA binding was the lowest in the control PBS formulation. In the HCE-T model, the elution kinetics of Na-HA were different between the tested eye drops. While most of the eye drops showed a sigmoidal increase in Na-HA, suggesting pronounced binding, Hyaluron-ratiopharm^®^ eye drops exhibited a pattern similar to the elution profile of the PBS control. In addition, the HCE-T model, when used in the declining channel set-up, provided the first simple evidence that allowed us to evaluate reformulations of eye drops. Over the years, changes in buffer media have likewise occurred, such as in Bepanthen^®^ eye drops (phosphate buffer) and BLUpan^®^ medical UD (citrate buffer), without any further modifications. Biological responses were evaluated by spreading experiments performed on synthetic surfaces. Clinical observations of corneal calcification that was attributed to the presence of phosphate within eye drops (*i.e.*, in patients treated for dry eyes) have been characterized [67] in *ex vivo* Eye Irritation Tests (EVEITs). Previous clinical observations showed that the topical use of artificial tears containing phosphate in injured eyes led to sight-threatening corneal complications in high-risk patients. By simulating these treatment conditions, the EVEIT convincingly demonstrated that changes in the composition of pharmaceutically administered treatments, similar to switches in the buffer from phosphate to citrate buffer, can prevent this undesired side effect. Eye drops containing citrate buffer did not cause corneal calcification in the eyes.

Several parameters and their combined impact on binding in the different *in vitro* setting should be mentioned. Changes in parameters might be driven by natural circumstances in the *in vitro* models or the experimental set-up conditions. On the one hand, differences might be due to differences in the *in vitro* structure of the corneal surface used in HCE-T and that observed in *ex vivo* pig corneas. Although they were highly correlated [16], the HCE-T monolayer had a less slippery surface structure than was found on *ex vivo* corneas. Second, differences in incubation times, periods and handling (stirring, pipetting, *etc.*) can affect results. Because the binding capacity of Na-HA is low, these parameters, although kept constant within the assays, might have impacted the results. The most moderate set-up, the HCE-T monolayer, when used with a flow rate of 18 µL/min, produced higher bindings. A third parameter, the proportion of the *in vitro* model surfaces and the applied volumes, was also different across the conditions. For example, 400 µL of 3H-Na-HA-spiked eye drops or PBS were applied to a 0.64 cm^2^ surface in the FC and pig corneas, whereas in the HCE-T monolayer set-up, 35 µL was applied to a surface area of 9 cm^2^ (50 mm x 18 mm). In the declining channel method, only approximately 80% of the monolayer could interact with this volume, so that the experimental area was decreased to 7.2 cm^2^. In the FC experiments, 1 cm^2^ was exposed to a volume of 625 µL, whereas in the HCE-T model, 1 cm^2^ was exposed to a volume of 5 µL. Consequently, there were differences in the areas of corneal surfaces that could bind the formulations.

Our novel *in vitro* binding methods are valuable supplements to well-known physical tests of spreading and rheology. Additionally, the data obtained can be linked to the interplay with the corneal surface to fill in the gaps in the cornea in *in vitro* permeability tests [6]. *In vitro* corneal retention studies provide techniques that can provide further support within early formulation development and to classify mucoadhesive polymers [68], their mixtures and interactions of actives in ocular drug delivery.

## CONCLUSION

Both *in vitro* methodologies produced comparable evidence and rankings based on the determination of the rate of Na-HA binding to ocular surfaces in eye drops. In most cases, eye drop solutions enabled a higher extent of Na-HA binding than was observed in the PBS control. In general, the bindings were weak and might be more precisely described as retention. Furthermore, the HCE-T monolayer model provided a functional *in vitro* approach for determining the binding kinetics of Na-HA. Moreover, this method allows researchers to mimic lacrimation by using physiological levels of ATF.

The results of our *in vitro* studies enhance our knowledge about the capacity of Na-HA in eye drops to bind to the ocular surface. However, the *in vivo* relevance of these results should not be over-estimated. In fact, the *in vitro* designs depicted here represent important steps that support the development of eye drops that are to be applied to the corneal surface. However, their clinical significance is limited due to, for example, missing eyelid movements, varying dilutions on the surface and the lack of lacrimation. The HCE-T *in vitro* kinetics model might be a useful tool for studying the effects of formulations on corneal binding during drug development. Likewise, as was demonstrated for Bepanthen^®^ eye drops, which contain a phosphate buffer, *versus* BLUpan^®^ medical UD, which contains a citrate buffer, the *in vitro* binding assays demonstrated the potential to test the extent of equivalence with regard to binding kinetics. Finally, spreading experiments performed on glass and polyethylene terephthalate demonstrated the power of these tests to explore non-biological effects.

Within the development of locally acting eye formulations, a novel *in vitro* set-ups could be used as valuable discriminators to assess the effects of, for example, changes in preservatives or in the classification of new excipients with adhesive properties. In the future, it would be of interest to further correlate *in vivo* binding results to optimize experimental designs.

## Figures and Tables

**Table 1:** Summarized contact angles recorded on glass and PET surface. Mean data and standard deviations were calculated from experiment performed in six-fold.

**Eye Drop**	**Glass**		**PET**	
	**Mean [°]**	**SD [°]**	**Mean [°]**	**SD [°]**
HYLO-COMOD^®^ (Lot 287356)	46.9	1.5	109.5	2.0
Hyaluron-ratiopharm^®^ eye drops (Lot R10824)	48.9	5.0	112.1	1.2
Bepanthen^®^ eye drops (Lot 150548)	42.9	1.0	105.7	1.2
BLUpan^®^ medical UD (Lot 151286)	44.8	3.1	106.4	2.1

**Table 2:** Adjusted *p*-values for all pairwise comparisons between contact angles for glass and PET surface, respectively. Significant *p*-values (*p*_adj_ < 0.05) are underlined.

**Comparison**	**adjusted *p*-values**	
	**Glass**	**PET**
HYLO-COMOD^®^ - Hyaluron-ratiopharm^®^	1.000	0.918
HYLO-COMOD^®^ - Bepanthen® eye drops	0.120	0.183
HYLO-COMOD^®^ - BLUpan® medical UD	1.000	0.725
Hyaluron-ratiopharm® - Bepanthen® eye drops	0.096	0.002
Hyaluron-ratiopharm® - BLUpan® medical UD	0.918	0.017
Bepanthen^®^ eye drops - BLUpan^®^ medical UD	1.000	1.000
